# High-quality genome assembly and annotation of five bacteria isolated from the Abu Dhabi sabkha-shore region

**DOI:** 10.1186/s12863-024-01246-6

**Published:** 2024-06-19

**Authors:** Beenish Sarfraz, Jean Tuyisabe, Louis De Montfort, Abdulrahman Ibrahim, Shamma Z. Abdulkreem Almansoori, Haya Alajami, Asma Almeqbaali, Biduth Kundu, Vishnu Sukumari Nath, Esam Eldin Saeed, Ajay Kumar Mishra, Khaled Michel Hazzouri, Raja Almaskari, Abhishek Kumar Sharma, Naganeeswaran Sudalaimuthuasari, Khaled M. A. Amiri

**Affiliations:** 1 Department of Biology, College of Science, United Arab Emirates University, P.O. Box. 15551, Al Ain, UAE; 2 Khalifa Center for Genetic Engineering and Biotechnology, United Arab Emirates University, P.O. Box. 15551, Al Ain, UAE

**Keywords:** *Bacillus spizizenii*, Illumina, MinION, *Priestia Aryabhattai*, *Pelagerythrobacter marensis*, Salt flat, *Staphylococcus capitis*

## Abstract

**Objectives:**

Sabkhas represent polyextreme environments characterized by elevated salinity levels, intense ultraviolet (UV) radiation exposure, and extreme temperature fluctuations. In this study, we present the complete genomes of five bacterial isolates isolated from the sabkha-shore region and investigate their genomic organization and gene annotations. A better understanding of the bacterial genomic organization and genetic adaptations of these bacteria holds promise for engineering microbes with tailored functionalities for diverse industrial and agricultural applications, including bioremediation and promotion of plant growth under salinity stress conditions.

**Data description:**

We present a comprehensive genome sequencing and annotation of five bacteria (kcgeb_sa, kcgeb_sc, kcgeb_sd, kcgeb_S4, and kcgeb_S11) obtained from the shores of the Abu Dhabi Sabkha region. Initial bacterial identification was conducted through 16 S rDNA amplification and sequencing. Employing a hybrid genome assembly technique combining Illumina short reads (NovaSeq 6000) and Oxford Nanopore long reads (MinION), we obtained complete annotated high-quality gap-free genome sequences. The genome sizes of the kcgeb_sa, kcgeb_sc, kcgeb_sd, kcgeb_S4, and kcgeb_S11 isolates were determined to be 2.4 Mb, 4.1 Mb, 2.9 Mb, 5.05 Mb, and 4.1 Mb, respectively. Our analysis conclusively assigned the bacterial isolates as *Staphylococcus capitis* (kcgeb_sa), *Bacillus spizizenii* (kcgeb_sc and kcgeb_S11), *Pelagerythrobacter marensis* (kcgeb_sd), and *Priestia aryabhattai* (kcgeb_S4).

## Objective

Sabkhas, also known as salt flats, represent polyextreme environments with high temperatures, salinities, and light intensities and are distributed globally in arid regions of the Middle East, North Africa, the USA, and Australia. Sabkhas pose a challenging environment for the survival of plants, animals, and various organisms due to their extreme conditions [1, 2]. Despite the harsh environmental conditions, these salt flats host remarkably robust and diverse microbial communities that are highly adaptable and metabolically diverse and have excellent abiotic stress resilience [3–5].

Previously, our unprecedented research effort cataloged the rich microbial diversity and distribution dynamics of the Abu Dhabi sabkha region using a combination of 16 S rDNA profiling and whole genome metagenomic approaches [6]. However, there is a paucity of high-quality complete genome sequences of bacteria isolated from the Abu Dhabi sabkha region. Consequently, in this study, we present complete genome sequences and gene annotations for five bacterial isolates isolated from the Abu Dhabi sabkha-shore region that exhibit higher salt tolerance. The genomic resources and datasets generated in this study will serve as a valuable repository for exploring genes and pathways associated with abiotic stress tolerance as well as understanding the mechanisms that bacteria use to survive in extreme environments. Nevertheless, the information gleaned from these bacterial species could be exploited for comparative genomics research programs and pave the way for engineering microbes endowed with high plant growth promotion activity for enhanced performance under high salt-stress conditions, opening up new avenues for sustainable agriculture for feeding burgeoning population.

## Data description

### Methodology

The five bacterial isolates used for whole-genome sequencing (WGS) were isolated from soil samples collected from the Abu Dhabi sabkha-shore region. Details on the systematic sample collection, bacterial culture strategy, and storage procedure are described in our previously published report [6]. A snapshot of our data analysis workflow is presented in Table 1 (Data file 1).

High-quality DNA isolation, quantitation, quality checks and 16S rDNA-amplicon-based bacterial species identification were carried out according to our previously published methods [7]. Furthermore, bacterial isolates were identified as *Staphylococcus capitis* (kcgeb_sa; 100% identity and E-value = 0), *Bacillus spizizenii* (kcgeb_sc; 99.5% identity and E-value = 0), *Pelagerythrobacter marensis* (kcgeb_sd; 100% identity and E-value = 0), *Priestia aryabhattai* (kcgeb_S4; 98% identity and E value = 0) and *Bacillus* genus (kcgeb_S11; 97.53% identity and E value = 0) by amplifying and sequencing the complete 16S rRNA gene sequence (~ 1.5 kb) using the universal primers 27 F and 1492R.

For WGS, shotgun and long-read libraries were prepared as previously described [7] and sequenced on an Illumina NovaSeq 6000 (PE reads, 150 bp) and MinION, respectively. The genome sequencing read statistics generated for each isolate are summarized in Data file 2 (Table 1). Trimmomatic v.0.39 [8] was used to trim low-quality bases and adapters from the raw Illumina reads, whereas ONT-MinION reads were error corrected and trimmed using the CANU program [9]. A hybrid genome assembly was used to assemble whole genomes of bacteria using Unicycler pipeline [10]. The assembled genomes were polished with Illumina and ONT reads using Pilon v. 1.23 [11]. Plausible plasmid sequences were extracted from the genome assembly using a homology-based approach. In addition, the assembled sample species were confirmed based on the average nucleotide identity (ANI) method [12]. The gene predictions and annotations of the assembled genomes were performed using the Prokka/ NCBI-PGAP tools [13, 14].

Our hybrid assembly strategy produced a gap-free, high-quality single circular genome for all five bacterial isolates. The kcgeb_sa isolate identified as *Staphylococcus capitis* had a genome size of 2,471,401 bp (G + C: ~33.1%), a BUSCO score of 100% and 2484 genes including 2340 protein-coding, 63 tRNA, 22 rRNA, and 5 ncRNA genes and two plasmids of 47,919 bp and 3530 bp (Table 1, Data files 3, 4, 5, 6 and 7).

The isolate kcgeb_sc was identified as *Bacillus spizizenii* with a genome size of 4,130,445 bp and a G + C percentage of ~ 43.9%, a BUSCO score of 100% and 4179 gene models, including 3963 protein-coding, 86 tRNA, 30 rRNA, and 5 ncRNA genes (Table 1, Data files 8, 9 and 10).

The isolate kcgeb_sd was identified as *Pelagerythrobacter marensis* with a genome size of 2,902,066 bp (G + C: ~66.38%), a plasmid sequence (7769 bp), a BUSCO score of ~ 98.4% and 2774 genes, including 2728 protein-coding, 46 tRNA, 3 rRNA, and 3 ncRNA genes (Table 1, Data files 11, 12, 13 and 14).

The isolate kcgeb_S4 was identified as *Priestia aryabhattai* with a genome size of 5,052,464 bp (G + C: ~38%), a BUSCO score of ~ 93.5%, 5247 genes with 5056 protein-coding, 37 rRNA, 99 tRNA and 8 ncRNA genes (Table 1, Data files 15, 16 and 17).

The isolate kcgeb_S11 was identified as *Bacillus spizizenii* with a genome size of 4,130,172 bp (G + C: ~43.9%), a BUSCO score of 100% and 4178 genes with 3962 protein-coding, 86 tRNAs, 30 rRNAs, and 5 ncRNAs genes (Table 1, Date files 18, 19 and 20).

**Table 1:** Overview of the data files/datasets

Label	Name of data file/dataset	File types (file extension)	Data repository and identifier (DOI or accession number)
Data file 1	Data analysis workflow used for whole genome sequencing of bacterial isolates	PDF	Figshare: 10.6084/m9.figshare.25816543.v1 [15]
Data file 2	Raw data (Illumina and MinION) details	Excel	Figshare: 10.6084/m9.figshare.25838296.v1 [16]
Data file 3	*Staphylococcus capitis* (kcgeb_sa) genome assembly and annotation statistics	Excel	Figshare: 10.6084/m9.figshare.25975564.v1 [17]
Data file 4	NGS data for *Staphylococcus capitis* (kcgeb_sa)	Web link	NCBI data: http://identifiers.org/insdc.sra:SRP378207 [18]
Data file 5	Genome sequence of *Staphylococcus capitis* (kcgeb_sa)	Web link	NCBI data: http://identifiers.org/insdc:CP145595.1 [19]
Data file 6	Plasmid sequence of *Staphylococcus capitis* (kcgeb_sa)	Web link	NCBI data: http://identifiers.org/insdc:CP145596.1 [20]
Data file 7	Plasmid sequence of *Staphylococcus capitis* (kcgeb_sa)	Web link	NCBI data: http://identifiers.org/insdc:CP145597.1 [21]
Data file 8	Whole genome statistics of *Bacillus spizizenii* (kcgeb_sc)	Excel	Figshare: 10.6084/m9.figshare.25557906.v1 [22]
Data file 9	NGS data of *Bacillus spizizenii (kcgeb_sc)*	Web link	NCBI data: http://identifiers.org/insdc.sra:SRP377107 [23]
Data file 10	Genome sequence of *Bacillus spizizenii (kcgeb_sc)*	Web link	NCBI data: http://identifiers.org/insdc:CP145137.1 [24]
Data file 11	Whole genome statistics of *Pelagerythrobacter marensis* (kcgeb_sd)	Excel	Figshare: 10.6084/m9.figshare.25557891.v1 [25]
Data file 12	NGS data of *Pelagerythrobacter marensis* (kcgeb_sd)	Web link	NCBI data: http://identifiers.org/insdc.sra:SRP377106 [26]
Data file 13	Genome sequence of *Pelagerythrobacter marensis* (kcgeb_sd)	Web link	NCBI data: http://identifiers.org/insdc:CP144918.1 [27]
Data file 14	Plasmid sequence of *Pelagerythrobacter marensis* (kcgeb_sd)	Web link	NCBI data: http://identifiers.org/insdc:CP144919.1 [28]
Data file 15	Whole genome statistics of *Priestia aryabhattai* (kcgeb_S4)	Excel	Figshare: 10.6084/m9.figshare.25557897.v1 [29]
Data file 16	NGS data of *Priestia aryabhattai* (kcgeb_S4)	Web link	NCBI data: http://identifiers.org/insdc.sra:SRP489214 [30]
Data file 17	Genome sequence of *Priestia aryabhattai* (kcgeb_S4)	Web link	NCBI data: http://identifiers.org/insdc:CP145138.1 [31]
Data file 18	Whole genome statistics of *Bacillus spizizenii* (kcgeb_S11)	Excel	Figsahre: 10.6084/m9.figshare.25557900.v1 [32]
Data file 19	NGS data of *Bacillus spizizenii* (kcgeb_S11)	Web link	NCBI data: http://identifiers.org/insdc.sra:SRP489215 [33]
Data file 20	Genome sequence of *Bacillus spizizenii* (kcgeb_S11)		NCBI data: http://identifiers.org/insdc:CP145722.1 [34]

### Limitations

We used a hybrid genome assembly method with high-coverage WGS data (both long and short reads) to produce a gap-free, high-quality single circular genome from all the bacterial isolates. In addition, we used Illumina and ONT-MinION reads to error-correct and polish the assembled genomes, and the Benchmarking Universal Single-Copy Orthologs (BUSCO) v.4.1.4 [35] tool was used to assess the completeness of the final genome assemblies, which confirmed genome assembly completeness. As a result, the authors are unaware of any limitations in their genome assembly and annotation approaches.

Nevertheless, this data note focuses on the description and annotation of high-quality genomes of five bacteria isolated from the Abu Dhabi sabkha-shore region. More in-depth research is needed to understand the phylogenetics, gene functions, and metabolic pathways, as well as the distinct biosynthetic gene clusters associated with these bacterial isolates that allow them to survive in harsh environments.

## Data Availability

The data generated during this study have been deposited in the NCBI-SRA and NCBI-GenBank databases. The raw data generated for this study can be accessed through the SRA-BioProjects (accession numbers: PRJNA1075203, PRJNA1075202, PRJNA842421, PRJNA842422, and PRJNA842419) and SRA-BioSamples (accession numbers: SRP378207, SRP377107, SRP377106, SRP489214 and SRP489215) databases. The assembled genomes and plasmids were deposited in the NCBI-GenBank database (accession numbers: CP145722, CP145138, CP144918, CP144919, CP145595, CP145596, CP145597, and CP145137). The data access links for all the data mentioned above are provided in Table 1.

## Abbreviations

ONT: Oxford Nanopore Technology
PE: Paired End
WGS: Whole Genome Sequencing
BUSCO: Benchmarking Universal Single-Copy Orthologs
